# Commentary: How Child's Play Impacts Executive Function-Related Behaviors

**DOI:** 10.3389/fpsyg.2016.00968

**Published:** 2016-06-23

**Authors:** Timothy Rice

**Affiliations:** Icahn School of Medicine at Mount SinaiNew York, NY, USA

**Keywords:** play therapy, executive functions, emotion regulation, defense mechanisms, psychoanalytic therapy

## Introduction

In a recent review Shaheen (2014) surveys evidence-based and popular programs that are designed to advance the development of executive function in children. In this commentary I propose that psychoanalytic psychotherapy with children is an additional program and therapeutic orientation that promotes executive function development.

In support of this proposal I review the executive function construct, the relationship of emotion regulation to executive function, and the observed similarities between the implicit branch of emotion regulation system and defense mechanisms (Rice and Hoffman, 2014). Elsewhere Rice (2016), I have suggested that systematic interventions upon children's defense mechanisms may promote the development of the implicit emotion regulation system. Because this program of interpretation of children's defenses against painful feelings is an acknowledged (and unacknowledged) central intervention in child psychoanalysis (Hoffman, 2007), it becomes possible to propose that psychoanalytic psychotherapy with children advances executive function development through its promotion of implicit emotion regulation capacities through the technique of defense analysis.

## Executive functions and children's play

Executive functions are a set of prefrontal cortex-dependent processes that facilitate the attainment of defined goals (Shallice and Cooper, 2011). The set of processes includes inhibitory control, attentional control, set shifting, working memory, problem solving, and planning.

Sheehan's review emphasizes the means by which structured rule-bound games may improve executive functions. The programs of Bodrova and Halperin (Bodrova and Leong, 2007; Halperin et al., 2013) which build upon Russian learning theory and biobehavioral development (Luria, 1973; Vygotsky, 1978) have attained a significant evidence basis toward improving executive functioning (e.g., Halperin et al., 2013). These programs include games such as “Simon Says” where the child must attend to commands and inhibit action when a command is not preceded by the words Simon Says, “Dance and Freeze” where the child must inhibit all actions on the word freeze and recommence when the music resumes, and “The Opposite Game” where the child must inhibit the commanded action and set-shift to do the opposite. These games' relevance to inhibitory control appears in their empirical evidence base (e.g., Healey and Halperin, 2012).

Programs which derive from educational kinesthesiology (e.g., Williams and Shellenberger, 1994) and rehabilitation science (e.g., Sohlberg and Mateer, 2001) have less robust data (e.g, May-Benson and Koomar, 2010). Major guidelines (e.g., Zimmer and Desch, 2012), though not discounting their value as part of a comprehensive treatment plan, warn that research is limited and inconclusive.

Thus, insofar as the executive functions studied include mostly explicit, cognitive-motor tasks, there is existing evidence to suggest that play therapies promote executive function development.

## Executive functions and emotion regulation

Like executive functions, emotion regulation is another prefrontal cortex-dependent process (Gross, 1998, 2013). It involves matching one's emotions to attain a defined goal. Some (Carlson and Wang, 2007) have already explored the connection of emotion regulation to executive functions in children.

A recent conceptualization of emotion regulation that defines both conscious, explicit and unconscious, implicit processes (Gyurak et al., 2011) yields two neural systems with distinct neural correlates (Etkin et al., 2015). Explicit emotion regulation involves modulation of limbic and visceromotor centers by prefrontal areas including the dorsal anterior cingulate cortex (dACC) and the dorsolateral PFC (dPFC), while implicit emotion regulation involves more ventral prefrontal areas including the orbitofrontal cortex (OFC), ventromedial PFC (vmPFC), and ventral anterior cingulate cortex (vACC). These findings mirror conceptualizations of executive functions as classifiable into “hot” and “cold” cognitive functioning (Zelazo and Carlson, 2012), with “hot” executive functioning sharing conceptual and neuroanatomic correlates with implicit emotion regulation.

## Implicit emotion regulation and psychoanalytic psychotherapy

The implicit emotion regulation construct overlaps with that of defense mechanisms (Rice and Hoffman, 2014). The traditional child psychoanalytic perspective understands defense mechanisms protect the child against unwelcome or painful affects (Bornstein, 1945, 1949; Becker, 1974; Hoffman, 2007). The interpretation of a child's defenses against unwelcome affects may promote development of the implicit emotion regulation system (Rice, 2016) and a subset of “hot” executive functions.

The interpretation of a child's defenses against painful affects is an experience-near technique that helps the child understand how feelings are avoided or expressed and enables the child's play to unfold. For example, often in children disruptive, oppositional, defiant, and provocative behaviors serve to distance the child from bearing uncomfortable feelings. When these behaviors disrupt play a psychoanalytically-oriented clinician will comment on the disruption. This helps the child to reflect upon the meaning of his or her behavior. The child is helped to experience behaviors as protective against unwelcome feelings that may have emerged in the play. Helping the child to experience that these feelings need not be so scary and avoided allows the child to move the play forward. This enables the child to explore new and more adaptive means of experiencing uncomfortable affects. With a less rigid reliance on maladaptive defenses the child's capacity for more developed implicit emotion regulation and “hot” executive functions is advanced.

## Conclusion

Promoting the development of executive functions through its relation to implicit emotion regulation and defense mechanisms is a valuable and unique contribution of child psychoanalytic psychotherapy. This complements the predominant behavioral play interventions that focus upon explicit, cognitive-motor tasks.

The capability to operationalize defense analysis in a manualized intervention (Hoffman et al., 2015) offers a unique opportunity for contemporary child psychoanalysis. For example, Shaheen includes Stanley Greenspan's “Floor time” model (Greenspan and Wieder, 1998) in her review owing to its goal of advancing emotional development, yet she notes that this program, which she specifies was derived from a child psychoanalyst, was hindered in development of an evidence basis through the difficulty in operationalizing due to “individualized, child-centered nature of the timing and content of interventions.” The capability to integrate defense analysis with emotion regulation, executive functioning, and affective neuroscience has the opportunity to overcome this traditional shortcoming of psychoanalytic psychotherapy and perhaps promote a return of the methods of this modality to a more central position in contemporary health care in the near future.

## Author contributions

The author confirms being the sole contributor of this work and approved it for publication.

### Conflict of interest statement

The author declares that the research was conducted in the absence of any commercial or financial relationships that could be construed as a potential conflict of interest. The reviewer DR and handling Editor declared their shared affiliation, and the handling Editor states that the process nevertheless met the standards of a fair and objective review.
